# The Christmas Dinner

**Published:** 1909-12-25

**Authors:** 


### The Christmas Dinner.

In the good old days there was no disagreement about diet. Every house became a Headlong Hall at Christmas time, and though people talked a hundred years ago of the return to nature, it was never interpreted as a return to nuts, for our relation to the anthropoid apes was not understood, and the return to nature was a theory, and a theory alone. It is not so any longer; the plum-pudding and the turkey are threatened with displacement, and a crowd of food faddists of every description gormandise on pepsins and peptones from every quarter. The curious thing about these people, however, is that while they have realised, according to their own standard, the value—nutritive or therapeutic—of every kind of food, they have never realised the therapeutic value of over-eating. It is absurd to call the Christmas dinner a feast unless we mean that we eat then more and better than usual. The gourmand and the gourmet each has his own share in the menu. It is a fact which no medical man of experience will deny that change of diet and an occasional excess may produce a healthy action on the system, and that the small boy whose button parts company on Christmas night with his waistcoat is the illustration of a scientific truth. The Christmas dinner, then, is as useful and beneficial as the cyclonic disturbances which are always rapidly advancing from the Atlantic. This is no doubt the reason why Christmas only comes once a year—the fast and the feast—anything, in fact, says history, but not "the normal regimen." It would be interesting to compare the Christmas menus of the faddists and the faithful.

Will the same genial laughter with which we picture Mr. G. K. Chesterton this evening light up our faces as we think of Mr. Eustace Miles? There is the tradition of a holy drunkenness on one side, and there is the novelty of a scientific sobriety on the other. Which of the two is more likely to be saying with poor Mr. Chadband, "O let us be joyful"—which of the two is more likely to be keeping the feast? We leave the answer to our readers, because there are some shy Puritan souls among us who never could and never would agree with Dr. Middleton that every man on occasion "should exult to dine." There can be no doubt that the teaching of Science in this matter is, like all great teaching, paradoxical—her norm is the swing of the pendulum, not the equilibrium of a stationary rod. Fruitarians and beef-eaters alike, however, should remember the great definition of a Christmas dinner which all schoolboys know—that while "enough is as good as a meal, more than enough is as good as a feast." That, at any rate, is the spirit in which the Christmas dinner must be approached, and its exuberance is the spirit which the Christmas dinner is intended to foster; though individuals will have their own ideas of how it should be carried out. Those of us who have a respect for old wines, for hocks that "have compassed age," are inclined too lightly to condemn water-drinkers as cheerless creatures without a pulse of generous enthusiasm. But we have only to call to mind the frantic ravings of the teetotalers, the total abstainers, and the haters of the iniquity of beer, to see that a prolonged course of water drinking produces very similar symptoms, hysteria, want of self-control, to those of alcoholism. So the spirit of science and of Christmas both combine to foster a broad human toleration, and we can avoid the bogs of alcoholism and teetotalism by keeping to the broad causeway which the Church has given us by the institution of periodic feasts and fasts, knowing that her action in so doing is based on the scientific teaching that health is an unstable equilibrium far too complex to be attained by any facile orthodoxy in matters of diet.

